# Osteopathy

**Published:** 1898-10

**Authors:** 


### *EDITORIAL NOTES AND COMMENTS.*

The Business office of THE JOURNAL is 305, 306 Fitten Building.

The Editorial office is Room 401, 402 Grand Opera House.

Address all Business communications to Dr. M. B. Hutchins, Mgr.

Make remittances payable to THE ATLANTA MEDICAL AND SURGICAL JOURNAL.

On matters pertaining to the Editorial and Original communications address Dr. Dunbar Roy, Grand Opera House, Atlanta.

Reprints of original articles will be furnished at cost price. Requests for the same should always be made *on the manuscript.*

We will present, post-paid, on request, to each contributor of an original article, twenty (20) marked copies of THE JOURNAL containing such article.

### OSTEOPATHY.

THE osteopaths are still increasing in numbers and daily converts are being added, if we may judge from some of the journals which are being published in the interest of osteopathy. However, this is not surprising, for there are thousands of people anxiously waiting for something new to "turn up" in order that they may rush in and give it a trial. We believe it was Barnum who said that "the American people love to be humbugged." Massage is not new, and yet the osteopaths put it in a disguised light by calling it another name. The Swedes are the best osteopaths in the world. It is really amusing to read what an over-enthusiastic convert sometimes writes about osteopathy. Here is a sample from one who has been greatly benefited. The patient, a lady, thus defines osteopathy: Technically, "Osteopathy is that science which consists of such exact, exhaustive and verifiable knowledge of the structure and functions of the human mechanism, anatomical, physiological and psychological, including the chemistry and physics of its known elements, as has made discoverable certain organic laws and remedial resources within the body itself by which nature, under scientific treatment peculiar to osteopathic practice, apart from all ordinary methods of extraneous artificial or medicinal stimulation, and in harmonious accord with its own mechanical principles, molecular activities and metabolic processes may recover from displacements, disorganizations, derangements and consequent disease, and regain its normal equilibrium of form and function in health and strength."

There you have a concise definition of osteopathy, and as clear as mud. The writer adds (in jest, of course): "This is a long sentence, but comprehensive." Still further, the writer, who has evidently given great study to the subject and has it under entire control, gives the method of treatment as follows:

"The patient places himself in the hands of an expert anatomist, otherwise he can't be worthy of his name. The sufferer is divested of the outer clothing covering the trunk (?) of the body. Then the operator with the tips of the fingers, that are trained to the utmost sensitiveness, examines the spinal column from the medulla oblongata to or below the lumbar region. To the layman it is astonishing to have the expert tell him or her the exact condition of every internal organ. The diplomate (the name given to the practitioner of osteopathy) has detected any pressure upon the nerves issuing from the vertebra, spinal curvatures or contractions of any kind; in fact, he has ascertained just the condition of the entire system. Internal difficulties can be diagnosed without the use of the X-ray, and the diseased parts relieved through treatment of the nerve centers governing the base (?) motor and sympathetic nerve centers of the system."

How beautiful and fairylike, yet how childlike and simple!

We trust these references may give to our readers a perfect understanding of the science of osteopathy.

